# Boundary-Work and the Distribution of Care for Survivors of Domestic Violence and Abuse in Primary Care Settings: Perspectives From U.K. Clinicians

**DOI:** 10.1177/1049732321998299

**Published:** 2021-03-22

**Authors:** Anna Dowrick, Gene Feder, Moira Kelly

**Affiliations:** 1 Queen Mary University of London, London, United Kingdom; 2 University of Bristol, Bristol, United Kingdom

**Keywords:** UK, primary care, general practice, professional boundaries, boundary-work, violence against women, gender-based violence, domestic violence and abuse, qualitative, interviews

## Abstract

Health care encounters are opportunities for primary care practitioners to identify women experiencing domestic violence and abuse (DVA). Increasing DVA support in primary care is a global policy priority but discussion about DVA during consultations remains rare. This article explores how primary care teams in the United Kingdom negotiate the boundaries of their responsibilities for providing DVA support. In-depth interviews were undertaken with 13 general practitioners (GPs) in two urban areas of the United Kingdom. Interviews were analyzed thematically. Analysis focused on the boundary practices participants undertook to establish their professional remit regarding abuse. GPs maintained permeable boundaries with specialist DVA support services. This enabled ongoing negotiation of the role played by clinicians in identifying DVA. This permeability was achieved by limiting the boundaries of the GP role in the care of patients with DVA to identification, with the work of providing support distributed to local specialist DVA agencies.

## Introduction

In this article, we explore professional boundaries in the context of the changing responsibility of health care professionals with regard to domestic violence and abuse (DVA). Expectations of health care professionals have changed following increasing societal recognition of DVA as a violation of human rights, development of interventions to address and prevent it, and recognition of the impact of abuse on health outcomes. National and international policy guidance increasingly mandates that clinicians identify patients who have experienced abuse and direct them toward specialist community support (García-Moreno et al., 2014; National Institute of Health and Care Excellence [NICE], 2016).

We examine the negotiation of the boundaries of health care professionals’ responsibilities with regard to DVA. We take as a case study a U.K. DVA training and support program—Identification and Referral to Improve Safety (IRIS)—that has been implemented nationally to improve the primary care response to women experiencing DVA. In doing so, we make two contributions. First, we make visible crucial work involved in identifying patients in need of DVA support in general practice. Second, we highlight that boundary negotiation is an ongoing, interactional process requiring a permeable boundary between professional groups.

## Background

### DVA and Primary Care

7.5% of women in the United Kingdom currently experience DVA (Office for National Statistics, 2019). DVA is defined as “any incident or pattern of incidents of controlling, coercive, threatening behaviour, violence or abuse between those aged 16 or over who are, or have been, intimate partners or family members regardless of gender or sexuality” (Home Office, 2016). Abuse can take many forms, including, psychological, physical, sexual, financial, and emotional abuse. It results from a mix of societal, community, and individual factors which create and reinforce unequal power relationships within interpersonal relationships (García-Moreno et al., 2014).

There is growing recognition in public policy and academic research of the health consequences of DVA (Campbell, 2002; World Health Organization, 2012). These include increased presentation in emergency departments (Warren-Gash et al. 2016), increased rates of abortion or miscarriage (Cook & Bewley, 2008), increased presence of any sexual health problem (Coker, 2007), negative impact on mental health (Trevillion et al., 2012), and increased substance misuse (Gilchrist et al., 2010).

International and national guidance gives health care professionals explicit roles in responding to the DVA experienced by their patients. A focus on identification and building connections with community support reflects a purposeful avoidance of medicalizing DVA (Sweet, 2015), encouraging clinicians to view DVA as a complex social issue rather than a medical problem to be “fixed” (Rittmayer & Roux, 1999). Globally, primary care teams have been identified as playing a key role in the health care response to DVA (Gear et al., 2016; Hooker et al., 2015; Prosman et al., 2014).

In the United Kingdom, community-based general practitioners (GPs) are the access point to all health services for non-emergency care. They have been identified by British policy makers as well-placed to initiate discussions about DVA (Department of Health, 2017; Home Office, 2016). There are three factors which contribute to this in a U.K. context: their historic professional role as having a more holistic and relationship-based approach to health care than other professions (Rudebeck, 2019), their interconnections with multiple different community and secondary care services (Feder, 2006), and because GPs have the most contact with the affected population than other health services (Richardson et al., 2002).

Current national guidance (NICE, 2016) makes clear that addressing DVA is a core responsibility of primary care teams. GPs are encouraged to have a low threshold for asking about DVA during consultations but are not required to routinely screen for DVA. Following a disclosure, clinicians have a responsibility to identify immediate safety concerns (for the victim and any children) and to direct patients toward appropriate specialist support.

Despite this mandate, conversations about DVA rarely take place in primary care settings. Reasons for this include that many clinicians lack confidence in both recognizing abuse and knowing how to directly raise the topic with patients (Ramsay et al., 2012), and may avoid these conversations for fear of causing offense or escalating violence (Waalen et al., 2000). It can be challenging for women to actively self-identify as having support needs and raise DVA with professionals. This is because perpetrators make victims feel worthless and undeserving of help, as well as blocking opportunities to disclose by attending appointments with women (Mackenzie et al., 2019).

In the rare instances when a patient does self-identify as needing support, clinicians struggle to know what to offer in the absence of knowledge about community resources, or trust in their quality and availability (Ramsay, 2002; Yeung et al., 2012). Moreover, women that do not leave abusive partners can face stigma from professionals who perceive an “ideal candidate” for support to be one who exits an abusive relationship (Zweig et al., 2002). Taken together, these factors contribute to ongoing silence about DVA in health care consultations, despite national policy explicitly calling on GPs to identify DVA in primary care settings.

### The IRIS Program

**Figure 1.**
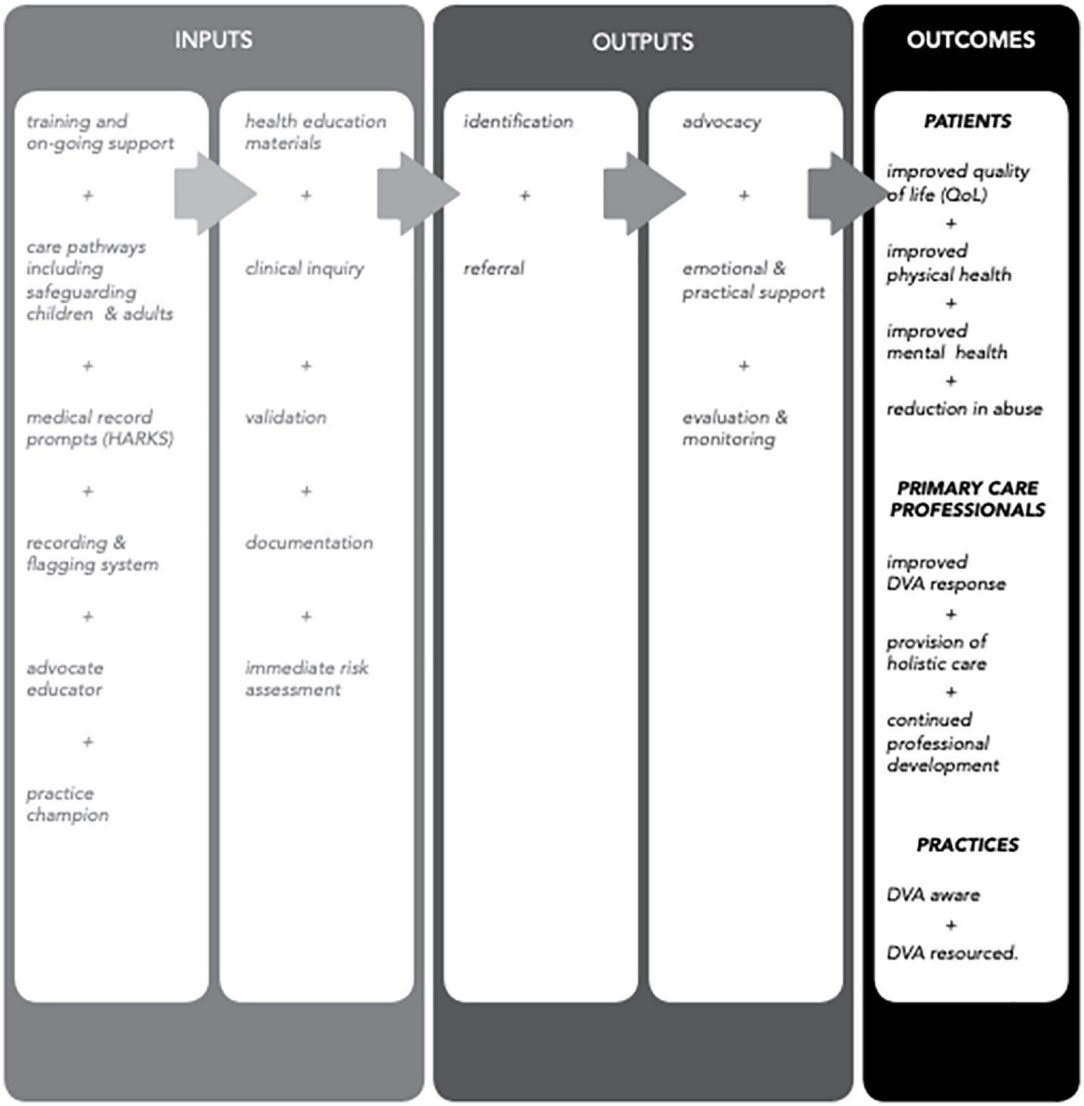
The IRIS model. *Source.* This figure is taken from a paper previously published by Dowrick, Kelly and Feder (2020). *Note*. DVA = domestic violence and abuse; IRIS = Identification and Referral to Improve Safety.

The IRIS program was developed in response to growing recognition that additional support was needed to enable GPs to engage in the work of addressing DVA in the United Kingdom. The model was designed based on emerging global evidence about DVA interventions and consisted of training for all clinical and reception staff, electronic prompts to ask about DVA during consultations (Sohal et al., 2007), and an ongoing connection for referral to a named specialist DVA worker (Advocate Educator) based in a local support agency. Full details of the program are reported in Figure 1.

The aim of the intervention was to increase both identification of DVA and referral for support. It sought to provide training to improve clinician confidence in how to recognize and safely ask about DVA and created direct referral routes between general practice and local support services.

Tested in a randomized controlled trial between 2007 and 2010, the IRIS program was successful at increasing conversations about DVA in primary care and increasing referrals to specialist support (Feder et al., 2011). Bridging funding enabled the model to be scaled up nationally from 2012 onward. IRIS is currently running in over 40 health care commissioning areas of the United Kingdom (IRISi, 2019) and is now delivered through an independent social enterprise (IRISi). It has been incorporated into U.K. national government policy on DVA (Home Office, 2016; NICE, 2016), It has also been adapted for other health care settings (Sohal, Pathak, et al., 2018) and other countries (Colombini et al., 2020).

While the IRIS program has been successful in improving identification and referral of cases of DVA, there has been minimal examination of *how* the intervention changes clinician practice. The purpose of the research this article reports on was to address this gap, conducted as part of a wider evaluation of the implementation of the IRIS program between 2015 and 2018. We asked the question, “How does the IRIS program change the practice of primary care professionals?” In this article, we further understand about the shifting boundaries of the role of GPs in relation to DVA, using the IRIS program as a case study.

### Shifting Professional Boundaries

The introduction of the IRIS program represented the first initiative in the United Kingdom to address DVA through establishing a structured connection between primary care and specialized DVA support services. This brought into contact two professional groups—GPs and DVA advocates—which previously had few interactions.

We interpret the building of connections between the two groups as a form of professional boundary-work, in which different groups undertake symbolic and material practices which enable demarcation of roles and responsibilities (Lamont & Molnár, 2002). In the context of DVA, this involves negotiating professional jurisdictions for the care and support of patients affected by DVA. This negotiation requires engagement with what is and what is not a legitimate part of GPs work with regard to DVA, which Fournier (2000) argues is a key element of boundary negotiation between professional groups. To understand what GPs consider to be legitimate work, we draw upon Suchman’s (1995: 574) definition of legitimacy as “the generalized perception or assumption that an entity’s actions are desirable, proper, or appropriate within some socially constructed system of norms, values, beliefs, and definitions.”

Nancarrow and Borthwick (2005) have offered valuable theoretical insight into dynamic shifts in the boundaries between health care professions, highlighting the potential for changes within roles (diversification or specialization) and between roles (substitution). The idea of diversification illuminates the work of expanding professional remits, such as the practice of multiple therapeutic approaches by primary care clinicians. Shuval et al. (2004, 2012), for instance, explored the way in which primary care clinicians navigate the boundaries between biomedicine and alternative therapies, highlighting the skill of negotiating institutional boundaries to legitimize their integrative care practices. Efforts to diversify the remit of GP roles to include directly supporting survivors of DVA have had limited success (Hegarty et al., 2010).

The substitution of tasks between professional groups has been examined extensively in relation to debates over jurisdictional shifts in task-based responsibilities, such as prescribing (Cooper et al., 2012) and workplace sickness certification (Welsh et al., 2014). Similarly, there has been significant study of disputes in decision making between professions with a shared jurisdiction over patients, for example, primary care and secondary care clinicians and between nursing and medical professions (Allen, 2001).

These studies emphasize that shifting the boundaries of professional roles is influenced by changing interactions and power balances between different professional groups, with debate over what constitutes legitimate work. Groups with the perceived lower status in a professional interaction seek that the actor with the perceived higher professional status substitutes tasks and decisions that confer authority. This enables role diversification and associated changes in status. Similarly, groups with higher status seek to substitute “dirty work” to others. Understanding these role changes in the context of boundary negotiations makes visible the work of maintaining, and resisting, dominance held by specific professions.

Research into boundary-work often frames negotiation in terms of contestation and dispute, building on Abbott’s (1988) seminal work on the formation of jurisdictions of different professions. Interactions between health care professions and community-based groups represent an opportunity to study jurisdictional responsibility differently, as these interactions take place outside of the hierarchical structures of health care professions. Our study explores the productive potential of boundaries for interaction and collaboration (Star & Griesemer, 1989). We examine boundary-work as a process of joint negotiation of responsibilities, highlighting interdependence rather than conflict between professions. We explore Nancarrow and Borthwick’s (2005) notion of *substitution*, where one group gives an aspect of their work to another group, in greater depth. In the context of DVA, clinicians are encouraged to substitute certain responsibilities by referring patients to specialist support services, creating a link between two previously unconnected jurisdictions.

To summarize, in this article, we focus on the specific role of GPs in the IRIS program. This builds on previous work examining how the IRIS program connects diverse professional interests in DVA (Dowrick et al., 2020). We make a step toward understanding the legitimacy of addressing DVA in general practice, exploring how U.K. GPs conceptualize and perform their responsibility with regard to DVA. In doing so, we explore how the IRIS program enables a renegotiation of the boundaries of GP roles with regard to the identification and support of patients affected by DVA.

We address two gaps in research. First, we add insights about boundary-work between primary care and community-based support services. Second, we examine the limitations of the idea of task substitution as a one-way or finite act, instead exploring substitution as a multidirectional action across permeable professional boundaries. We intend that the emerging insights will contribute to sociological understanding of the negotiation of shifting boundaries of professional roles in health care interactions, particularly with regard to partnerships with community-based services. We also intend that lessons from this work will support improvements in access to specialist support for women affected by DVA in the United Kingdom and beyond.

## Method

This study was conducted as part of a broader mixed-method investigation of the implementation of the IRIS program (Sohal, Feder, et al., 2018). The wider study aimed to improve understanding of the factors contributing to successful implementation of the IRIS program. This article reports on the qualitative aspect of this research program, which sought to understand *how* the IRIS program changed professional practice. At the time of conducting the research, the authors comprised AD (a female social science doctoral student), GP (a male academic GP), and MK (a female sociologist with background in nursing). GF led the original trial of IRIS and facilitated collaboration with the national IRIS implementation team, now a social enterprise, IRISi.

An ethnographic case study research design was adopted, with the rationale of enabling the team to develop detailed insights into the delivery of the IRIS program over time. Two geographic areas where the delivery of the IRIS program closely represented the original program model were selected as case studies. GPs from practices in these areas were invited to participate in interviews. This was with a view that they would be able to reflect on any changes in practice since the introduction of the program. Sampling had the aim of identifying clinicians who either frequently or rarely referred patients to specialist DVA services.

Practice managers were asked to distribute information about the interview study to allow the possibility of self-selection. Specialist support services were also asked to offer details of clinicians they knew who were either high or low referrers. Snowballing was done through recruited participants and local contacts known to the researchers.

Malterud et al.’s (2016) concept of *information power* was used to guide the sampling approach. Moving beyond the pervasive idea of saturation in qualitative research, they argue for a more nuanced focus on the quality of information within a sample. What might constitute sufficient information in a given project relates to the aims of the study, use of theory, specificity of the sample, quality of dialogue, and approach to analysis. For this study, we interpreted that we had enough information when different practices of engaging in DVA became visible, with clear description of how the IRIS program (among other factors) influenced these practices. We stopped recruiting when we had enough data to conduct a thorough analysis that addressed the emerging research questions.

A total of 13 interviews were conducted by AD. Participants ranged from 1 to 34 years in practice. Eight identified as female and five as male. All had attended at least one session of IRIS training. The range of time elapsed since training was between 6 months and 2 years. Since receiving training, seven had referred more than five patients to the IRIS service and six had referred one or none. Participants identified as White British (10) and British Asian (three). Interviews were done either face-to-face in general practice (seven) or by telephone (six).

Participants gave verbal and written consent of their participation at the start of the interview. Interviews were semi-structured, informed by an interview guide developed by AD. This guide drew on insights from research on the uptake of new practices in health care settings, focusing on how DVA was understood, what clinicians believed their role to be in addressing it, how they undertook the work of identifying and referring patients, and how they evaluated the outcomes of engaging in actions around DVA. The study guide was piloted with GPs known to the study team. All interviews were audio-recorded and transcribed using a transcription service and were supplemented by field notes written before and after each interview.

The authors adopted a pragmatist ontological approach, which posits that reality is continually produced through practice (Cornish & Gillespie, 2009). Informed by this, thematic analysis was undertaken (Braun & Clarke, 2006). This was with the intention that identifying themes relating to practice would give insight into how GPs construct their work and conceptualize the place of DVA within this.

There were two stages to the analytical process. The first stage of coding was undertaken to generate a broad understanding of the content of the data with types of practice within the data and how they related to one another (Silverman, 2010). AD familiarized themself with transcripts by reading them in full and listening back to recordings. Analysis was paper based, with initial notes written directly onto transcripts. Codes were collected together on a cover note for each transcript. Potential themes across all the transcripts were collated separately as the analysis developed. This process was guided by Miles and Huberman’s (1984) approach, with the following concurrent flows of activity: data reduction (focusing and simplifying data), data display (organizing data in a way that permits conclusions to be drawn), conclusion drawing (deciding what things mean), and verification (confirming provisional conclusions across the data).

After generating a broad overview of the data through the first stage of analysis, the authors decided on boundary practices as a focus for further detailed analysis. This was both inductive, in that the authors interpreted that the negotiation of jurisdictional boundaries was a key issue for participants, and deductive, in that the second stage of analysis was explicitly informed by further engagement in the theoretical literature on boundary-work. In particular, we drew on Nancarrow and Borthwick’s (2005) theory of dynamic professional boundaries as a heuristic with which to interpret the data.

The second stage of analysis followed the same process, but with a specific focus on coding talk associated with the boundaries of GP’s roles in relation to DVA. Having analyzed all the transcripts individually, AD employed the One Sheet of Paper (OSOP) approach (Pope et al., 2000; Ziebland & McPherson, 2006). Codes from different transcripts were grouped and displayed together using the OSOP method and regularly reconfigured until the arrangement enabled the development of a clear thematic narrative. The authors met regularly to discuss the process and to clarify any disagreements over interpretation of the data. Member feedback sessions were held with members of the IRIS delivery team in each case study area. All authors contributed to the development of the article.

This study received ethics approval from the Queen Mary Research Ethics Committee (reference: QMERC2015/29a and QMERC2015b), the Barts Health Joint Research Management Office (ReDa Number: QMERC2015.29b), and the appropriate local National Health Service (NHS) governance bodies.

## Findings

In the following sections, we detail two forms of work that participants described regarding how they distribute responsibility for addressing DVA. First, we draw attention to the work of distributing responsibility for the provision of support to specialist services. Second, we highlight how this substitution of work enables GPs to take additional responsibility for making space for conversations about DVA during consultations.

### Distribution of Responsibility for DVA Support

#### Clarifying the role of GPs in DVA support

Participants in this study recognized that DVA had a bearing on their professional role as GPs. They perceived connections between DVA and the overall management of their patients’ health. One participant articulated this clearly:

I enjoy general practice because we do take a holistic view and it’s not just about the medical side of things, it’s also about the social and the psychological side of things. You know, everything suffers if somebody is undergoing domestic violence, so you can’t really separate that out from their full control of diabetes, or their fatigue or general tiredness.

While acknowledging that understanding DVA was a legitimate aspect of addressing the health of patients, this participant and others felt discomfort holding the responsibility for providing DVA support to patients. While DVA was acceptably within their professional jurisdiction as a social determinant of health, they felt they lacked the ability to take actions to resolve DVA. Another participant explained how this made clinicians feel uncomfortable:

The thing I like about IRIS is it gives you the next step, because otherwise you talk to somebody, I’m talking to you and then you think, “I don’t know what to do!” And then you may miss that one opportunity to help.

Participants expressed that before becoming involved in the IRIS program, they felt “a bit lost” about what to do with a disclosure and would “scrabble” to find somewhere to refer the patient. One GP described the experience,

There’s definitely a fear thing and so I think that would put people off. I think it’s about teaching doctors “it’s OK,” and that there are then things you can do with that information. You don’t just have to think, “oh, my goodness me, what on earth do I do now?”

The ambiguous work associated with *what on earth do I do now?* was what clinicians sought to substitute. As generalists, GPs regularly participate in boundary-work to determine the limits of their field of practice in collaboration (and contestation) with specialist services to meet needs that fall outside of their expertise. Clinicians felt uneasy asking about DVA and anxious upon receiving disclosures in the absence of a clear understanding of their professional jurisdiction.

#### Reconfiguring the work of addressing DVA

With the introduction of the IRIS program, clinicians were given an overview of what could be done to support patients experiencing DVA through training delivered by Advocate Educators. Moreover, they were given tools to guide and shape the consultation: examples of validating responses that they could give to patients and a direct referral pathway to a named specialist who could identify a patient’s support needs and liaise with other services. A participant described how this changed her approach to DVA:

Just thinking about even asking the question, because it probably wasn’t on my radar as something to ask until the training. And then, yeah, knowing a bit about what exists so you can explain different options to them. And being able to say to them, “Actually, you’re not alone, many people go through this.” I think that’s quite helpful.

The opportunity to refer addressed clinicians’ concern about being able to take an action in relation to DVA. While clinicians in this study broadly conceived of DVA as being relevant to their role, they did not want sole responsibility for addressing it. Another participant reflected on how referral enabled him to substitute the responsibility for “solving the problem” to another service:

It’s no good me asking all those questions if all I can say to this lady is “Thank you for telling me.” There’s a therapeutic role, but what it’s going to generate is she’s going to come back and talk to me about it again and again and again, and that will make her feel better for the five minutes she’s here and maybe she’ll know that somebody is caring that she’s shared it with, but we haven’t really helped in terms of trying to solve her problem. We’ve helped her to cope with it maybe a little bit, but having IRIS means that (a) you can identify it and (b) you’ve got a referral pathway.

While recognizing that he is able to help a patient *cope with it*, he emphasizes that they do not succeed in the work of *trying to solve her problem*. Moreover, he was concerned that a patient would come back *again and again and again*. The introduction of a referral pathway puts a boundary on his interaction with a patient. It clarifies his role as limited to identification and enables him to substitute the work of *trying to solve her problem* to another profession, in this case a DVA specialist, via a referral pathway.

#### Patient engagement in distributing support

While the IRIS service offered a potential for distribution of work, it was not guaranteed. First, patients might disclose DVA but not want a referral to support services. For women at early stages in their recognition of abuse, simply being heard and validated by clinicians may be enough for them. Participants understood that a patient might disclose simply to *get it off her chest*:

It was almost as if she was just telling me because she wanted to get it off her chest and wanted me to know why she was so anxious and depressed, but without necessarily actually wanting to do anything about it.

Identifying DVA in primary care meant formal recognition that a patient had a need for support. If patients did not want a referral, GPs were not able to substitute the responsibility for addressing ongoing safety to other professionals. Instead, this created a responsibility for the clinician to ensure that they were safe. Another participant articulated what this meant for her:

If somebody has decided not to take that help, when they’ve disclosed something, that would automatically again raise to the forefront of, well, we need to make sure this person is all right every time they come in now.

Second, support offered might not meet the needs of patients. A GP described a challenging case of this:

In the end, now a year on [after referral to IRIS], the lady has gone back to her partner, because she didn’t think that her alternative, what she was offered residentially, was good enough for her child and baby. So actually, it’s been a big serious incident now, her partner has now got the solicitor to write a letter to the GP who referred her saying that everything that was documented was wrong, and she had to reply. So when it goes wrong, it does go really wrong and that’s a lot to deal with for some of the doctors. it just makes them think, “hang on, these services are not working, why am I getting involved in the whole thing, should I really have put that much effort in!?”

Her reflection—*should I really have put that much effort in?*—demonstrates that engaging in the work of addressing DVA involves ongoing evaluation on the perceived investment made by others in the wider network of DVA support. In this case, the lack of appropriate options enabling long-term change, and the efforts of perpetrators against the recognition of DVA by clinicians, led her to question the worth of the work being done in general practice.

#### Sharing care across permeable boundaries

The IRIS service offered support not only to patients but to clinicians making referrals. A participant reflected on how contact with IRIS made both parties feel supported.

Firstly, it makes the clinician feel better if you know that there’s something in place, so you’re not managing the burden alone. Maybe, I would imagine, it empowers the woman, because even if she decides to stay, she’s not taking the burden anymore.

Clinicians found it easier to remain engaged with DVA and to maintain optimism about outcomes if they felt that care was a joint effort with other professionals. They wanted to feel that they were not *managing the burden alone*. Another GP elaborated on this:

The feedback that you got from the person that was dealing with it from IRIS was what made it feel like it was more like a partnership and it was work in progress, rather than seeing things as success or failure, or even “do the referral, problem solved!” I think often with these cases, it’s not like that because resolution, if it happens at all, often might happen quite a bit further down the line. I think just sharing the burden with someone, or feeling that it’s more of a team effort I think is quite helpful, because otherwise it can feel like lots of things, but dispiriting is one of the things and frustrating.

In this example, he emphasizes the permeability of the boundary with IRIS practitioners. While the responsibility for *dealing with it* was distributed to the IRIS service, both parties retained an ongoing relationship with patients and bore witness to changes in experiences of DVA as *work in progress* over time. The work remained a shared jurisdiction. Given this, a permeable boundary allowing them to share the emotional burden of care was crucial. Moreover, the permeability of the boundary worked in both directions. GPs were able to reconnect with the IRIS service over time, and the DVA advocates were also able to contact GPs with concerns about violence-related health problems. A participant, reflecting on the value of the service, offered this insight:

I guess that’s what the IRIS service offers, the confidence that there is somebody or people there who can help.

In summary, trusting that reliable, accessible support was available was a critical step in enabling clinicians to start conversations about abuse and identify candidates for DVA support. Some practical aspects of care were substituted, whereby the IRIS service took responsibility for addressing the patient’s DVA support needs. This put a temporary boundary on the work of GPs and clarified and legitimized their role in identifying DVA. The opportunity to refer provided both a structure and a temporary closure to an interaction about DVA.

There is rarely a complete substitution in general practice. Many clinicians described navigating ongoing relationships with both survivors and perpetrators of abuse. Success in this ongoing work was judged in terms of maintenance of a permeable boundary with support services, with the practical and emotional work of care shared between multiple parties.

Having explored the distribution and negotiation of responsibility between primary care and specialist DVA support services in provision of DVA support, in the following sections we examine how clinicians present the distribution of responsibility for raising conversations about DVA between themselves and patients.

### Distributing Conversations About DVA

#### Creating space for conversations about DVA

While the IRIS program enabled clinicians to substitute aspects of the work of addressing DVA to other services, it encouraged them to take more responsibility for raising the topic of DVA in consultations. Before the introduction of the IRIS program, most participants in this study believed that the responsibility to raise the topic of DVA lay with patients. Clinicians highlighted a specific discomfort relating to inquiry about violence in relationships. This was linked to a concern that probing for this information was *too personal*. A participant elaborated on this:

Yeah, maybe they think you’re over-stepping the boundaries asking about something that’s too personal that they might not want to talk about.

Training received as part of the IRIS program emphasized that women want to be asked about DVA by a trusted professional, and that it was appropriate to ask directly. The training also highlighted connections between DVA and common presentations in general practice, such as anxiety, depression, sleep disorders, gastrointestinal dysfunction, and medically unexplained symptoms. This legitimized inquiry about the dynamics of relationships as an appropriate part of diagnostic work. A participant articulated this:

It’s not you being nosey or prying; “actually that could be something that affects your diagnosis and your treatment, and it’s important to know about it.”

Taking responsibility for actively engaging in patient narratives was considered an important route toward opening discussion of DVA. Another participant described her approach:

Picking up on little things; they’re often just little chance comments and if you ask a second question about it, then I think if people are given the space to talk, they will.

These accounts imply that the work of talking about DVA involved allowing space in a consultation for the patient narrative to emerge. A participant offered an example of how she unexpectedly received a disclosure. A patient had visited to discuss a pain in her elbow but then steered the conversation toward concern about bad breath.

While she was talking I was thinking, “wow, this is having a massive impact on her life, this is more than about bad breath,” and suddenly you get this flash of, “oh, I wonder what’s going on!?”

Upon exploring further, she discovered that the patient’s partner had been criticizing her breath, which led to a disclosure of abuse. The *flash* she describes resonates with a number of different accounts in this study, moments where clinicians realized that the consultation could take a different route. This realization might then redirect the consultation.

#### Building trust to enable a disclosure of DVA

Exploration did not guarantee a disclosure of DVA. Disclosure is a potential threat to safety, so patients have to feel confident that they will be understood and protected by a professional. Participants argued that clinicians who incorporated a consistent performance of empathy into their role were more likely to receive a disclosure. A participant reflected on this:

I think the way certain people practice, you attract certain types of cases, so if you’re seen to be a bit more empathetic, you probably attract people who are having a difficult time.

Another participant also highlighted the importance of building trust over time, and described how it might take several appointments with questions about DVA before a disclosure:

I think patients will choose a GP perhaps. Because they’re pretty good at sussing you out, patients. I think that’s why often it takes a few attendances or questioning before they disclose, they’re sort of checking you out.

As well as having insight into whether DVA might be an issue for a patient, clinicians also had to reflect on whether they had created the conditions under which a patient would feel safe to disclose. One participant asked herself the question “Do I feel like I’ve got a good enough relationship with this person that they’re going to disclose it to me?” before she asked anything of her patients.

#### Choosing not to ask about DVA

As well as being able to follow different routes through consultations to make space for a conversation about DVA, clinicians were also able to follow routes that could close this space down. One participant framed this choice in light of the challenge of being emotionally available for patients:

With a doctor you’re not getting the same person at the start of the day as you are at the end of the day. So the point is if you’re tired, if you’ve been speaking to people all day, your emotional reserves are much less at the end of the day, so what you have to offer patients, you just don’t have the emotional energy to do it. Because the point is making yourself emotionally available isn’t necessarily longer work, it’s a different type of work.

At times, when they were busy or tired, ignoring cues to discuss DVA was preferable for participants. Not asking about DVA meant they could avoid what one participant described as the work of “managing the answer”:

You’ve got to have the skills as well of how to raise it, but not only introduce it and raise it, but also then be able to manage the answer, whatever that might be, whether it’s surprise or shock, or upset or whatever.

Taking responsibility for engaging in the work of identifying candidates for DVA support necessitated being prepared to manage a range of responses from patients. Another GP, for example, feared a negative response if he raised DVA incorrectly:

If you get it wrong, the response is the wrong way round. It can backfire on you because that person will then go away thinking, “Well, I’m not going to see him again; he obviously thinks there’s something going on and there isn’t.” So that’s why it’s sometimes easier not to ask, because I haven’t got to pick up any pieces afterwards, I haven’t got to manage it at all.

In the case of a disclosure, he could substitute work to the IRIS service, but in this example, he acquires additional work of picking up the pieces of the relationship. Providing a good response, either to a disclosure or to shock at the question, was considered work.

Connection with the IRIS service encouraged GPs in this study to reflect on their responsibilities in making a disclosure possible. Before being able to substitute aspects of care to other services, they had to recognize and prioritize practices which would enable them to identify patients who need support. These included actively making space for narratives of abuse to emerge in consultations, and consistently presenting themselves as someone who could be trusted with a disclosure. Identifying a patient needing DVA support involved an ongoing commitment to creating the possibility of disclosure across multiple encounters with patients. In recognizing this as work, participants also acknowledged that there were instances where they avoided it. Constraints of time, energy, and perceptions of risks to patient rapport all played a part.

## Discussion

In this article, we have examined how U.K. GPs involved in the IRIS DVA training and support program account for changes in their professional practice in the context of DVA. We have argued that changing practice with regard to DVA involves negotiation and distribution of work in connection with other professions.

### Permeable Professional Boundaries

Nancarrow and Borthwick (2005) theorized two ways in which the boundaries of professional roles could change: diversification (taking on additional tasks) and substitution (giving unwanted tasks to other professions). In this study, we found that primary care clinicians were reluctant to diversify their roles to take on responsibility for addressing DVA but were comfortable to substitute this work to trusted specialist support services. However, this was not a total substitution. Patients remained within a shared jurisdiction of both primary care and DVA support services.

Our analysis of this shared jurisdiction has enabled expansion of Shuval et al.’s (2004, 2012) concept of permeable professional boundaries. Shuval et al. highlighted the importance of permeable boundaries in relation to the diversification of roles, identifying that they enabled primary care practitioners to strategically highlight or minimize their engagement with both biomedicine and alternative medicine. In this study, we found that permeable boundaries were also important in the context of role substitution.

Previous literature has established that clinicians are unwilling to engage in conversations about DVA if they feel a responsibility to “fix” it (Ramsay et al., 2012; Rittmayer & Roux, 1999), with engagement improved when GPs approach addressing DVA as a long-term process with no singular outcome (Hegarty et al., 2020). While GPs were reluctant to take further responsibility for addressing DVA alone, they reconfigured their responsibilities by putting in place a permeable boundary with specialist DVA support services. This enabled them to take ownership of aspects of care they felt comfortable with, such as creating space for patient narratives in consultations and monitoring health outcomes, while substituting overall responsibility for addressing a patient’s DVA support needs to other services.

Tasks were distributed between GPs and specialist DVA support workers, with ongoing interaction enabling jurisdictions to be clarified and adjusted relative to the uncertain trajectories of how a patient’s life might change in the context of DVA. Fournier (2000) argues that it is not the division of such labor itself that results in change in practice, but the interactions across boundaries of different professions that result in (re)negotiation of the competencies of different groups. Put another way, the interaction of different roles creates opportunities to clarify and contest responsibilities. For practitioners in this study, this was a repeated negotiation of responsibility, rather than a one-off substitution. In contrast with studies which demonstrate battles for jurisdiction between professions (such as Cooper et al.’s, 2012, examination of boundary-work between doctors, nurses, and pharmacists in relation to prescribing powers), this study highlights an interdependence of professions acting within a shared remit of care.

### Caring in the Context of DVA

As we have demonstrated, by introducing the ability to substitute work to the IRIS service, attention was drawn to the work required to identify potential candidates for support. This legitimized conversations about DVA in general practice and enabled GPs to reflect on their own skills at enabling narrative-focused, empathetic consultations and their value in enabling patients to disclose DVA.

Discussion of DVA was reframed as a style of consultation that emphasized engagement in the “lifeworld” of the patient (Barry et al., 2001). This reflects a movement toward trauma-informed care primary care, in which recognizing and addressing trauma is seen as important for overall health and well-being (Hamberger et al., 2019). As well as listening to and creating space for patient narratives, clinicians had to consistently present themselves as someone safe to disclose to. This can be characterized as a form of emotional labor (Hochschild, 1983), revealing that an important aspect of clinicians’ work is to elicit a feeling of safety in patients. There are costs for those undertaking emotional labor, commonly emotional burnout (Zenasni et al., 2012). Clinicians in this study identified these costs as a reason why they might avoid conversations about DVA.

Participating in the IRIS program enabled clinicians to see themselves as part of a wider network of DVA support. As well as connecting them with specialist DVA support services, they were given vision of other “planets” (Hester, 2011) within the ecology of DVA care, such as agencies with responsibilities for child protection, housing, and criminal justice. However, this improved connection also made visible the challenges and limitations of work within this network. Clinicians reported frustration when they perceived that other actors, such as housing agencies, were not doing the work expected of them. In particular, the ongoing withdrawal of public and community services to support survivors of abuse made efforts to support patients fraught.

A clear limitation of this research is that we did not gain insights from GPs who rejected the value of addressing DVA in primary care settings. We recommend that further research is conducted with clinicians who actively choose not to engage in discussions of abuse with patients. While this research was undertaken in a U.K. context, we believe that the findings have salience for a wider range of primary care settings globally.

## Conclusion

This study aimed to explore *how* connection with the IRIS program changed professional practice with regard to DVA in primary care settings. We have provided insight into how interactions across boundaries of primary care and community-based services clarify the jurisdiction of GPs in responding to DVA. We aimed to make visible the complex, interactional work of identifying patients as potential candidates for DVA support. We improve understanding of boundary-work, in particular the practices of task substitution. We present this work as an ongoing negotiation of shared responsibilities enabled by a permeable boundary between professional groups. This work is supported by programs such as IRIS, which empower GPs to both substitute aspects of the work of care to specialist services and be active in taking responsibility from patients in creating opportunities for disclosure.

A question often posed about GPs in the context of DVA is: *Why don’t they ask?* Having explored their accounts of the work of identifying candidates for DVA support, we suggest that a better question is: *Are there collaborative relationships in place which enable a safe discussion of DVA?* Our research indicates that two types of relationships might contribute to this: trusting relationships between general practice and specialist support services, and trusting, non-judgmental relationships between clinicians and patients.

Our findings have implications for both policy and practice. The precarity of funding for specialist DVA and affiliated services (e.g., legal aid and housing support) is an ongoing barrier to the development of sustainable relationships between primary care and DVA support services. Policy makers must enable formal, long-term connections between primary care and specialist community support services to maintain clinician engagement in DVA. With regard to practice, the increasing workload facing primary care limits opportunities for genuine and consistent engagement with patients. Addressing this involves consideration of the practical pressures faced by primary care and how the emotional labor of patient care can be shared within teams and between services.
